# Evaluating MRI correlates of lifestyle-based dementia risk reduction: Results from the AgeWell.de imaging study

**DOI:** 10.1177/13872877251414423

**Published:** 2026-01-13

**Authors:** Andrea E. Zülke, Frauke Beyer, Melanie Luppa, Toralf Mildner, Thomas Frese, Jochen Gensichen, Hanna Kaduszkiewicz, David Czock, Hans-Helmut König, Birgitt Wiese, Wolfgang Hoffmann, Jochen René Thyrian, Arno Villringer, Steffi G. Riedel-Heller, A. Veronica Witte

**Affiliations:** 1 Institute of Social Medicine, Occupational Health and Public Health (ISAP), Medical Faculty, University of Leipzig, Leipzig, Germany; 2 Department of Neurology, Max Planck Institute for Human Cognitive and Brain Sciences, Leipzig, Germany; 3 Bordeaux Population Health Research Center, University of Bordeaux, Inserm, UMR 1219, Bordeaux, France; 4 Institute of General Practice and Family Medicine, Martin-Luther-University Halle-Wittenberg, Halle, Germany; 5 Institute of General Practice and Family Medicine, University Hospital LMU Munich, Munich, Germany; 6 Institute of General Practice, University of Kiel, Kiel, Germany; 7 Internal Medicine IX – Department of Clinical Pharmacology and Pharmacoepidemiology, Heidelberg University Hospital, Heidelberg, Germany; 8 Department of Health Economics and Health Service Research, University Medical Centre Hamburg-Eppendorf, Hamburg, Germany; 9 MHH Information Technology – Science & Laboratory, Hannover Medical School, Hannover, Germany; 10 German Centre for Neurodegenerative Diseases (DZNE), site Rostock/Greifswald, Greifswald, Germany; 11 Institute for Community Medicine, University Medicine Greifswald, Greifswald, Germany; 12 Faculty V: School of Life Sciences, University of Siegen, Siegen, Germany; 13 Cognitive Neurology, University Hospital Leipzig, University of Leipzig, Leipzig, Germany

**Keywords:** Alzheimer’s disease, dementia, lifestyle, neuroimaging, prevention, risk factors

## Abstract

**Background:**

Multidomain lifestyle interventions can improve dementia risk by risk factor modification. Little is known about possible mechanisms underlying this effect.

**Objective:**

Analyze whether changes in a validated dementia risk score were linked to changes in neuroimaging markers in a sample of older adults at increased dementia risk, participating in a multimodal lifestyle intervention.

**Methods:**

Participants of the multi-centric AgeWell.de-trial at the Leipzig study site were examined using 3 Tesla MRI at baseline and 24-months follow-up, assessing markers of hippocampal-limbic atrophy and vascular pathology (hippocampal volume (HCV), entorhinal cortex thickness, free water fraction, peak width of skeletonized mean diffusivity, white matter hyperintensity volume, mean gray matter cerebral blood flow). Dementia risk was assessed using the Lifestyle for Brain Health (LIBRA)-index. Multivariable linear regression analyses assessed effects of changes in LIBRA on neuroimaging markers.

**Results:**

Of 56 participants at baseline, 41 underwent the follow-up assessment (M_age_: 68.1 (4.1), % female: 46.3, intervention/control group: 16/25). Lower LIBRA-scores, indicating lower dementia risk, were associated with higher HCV at baseline. LIBRA improved in both groups, with no between-group difference in change. Increases in LIBRA were linked to smaller decline in HCV independently of the intervention. No further effects of lifestyle changes on neuroimaging were detected. Exploratory analyses indicated that detrimental lifestyle changes were linked to decreased cognitive performance in the intervention group.

**Conclusions:**

We found no conclusive evidence for associations between lifestyle changes due to a multidomain lifestyle intervention and structural brain health markers. Larger samples and longer interventions may clarify underlying mechanisms.

## Introduction

Evidence on modifiable risk factors for Alzheimer's disease (AD) and dementia has been accumulating rapidly. The latest report of the Lancet Commission on Dementia Prevention, Intervention and Care suggested that 14 modifiable risk factors, encompassing physical inactivity, obesity, smoking, excessive alcohol use, diabetes mellitus, elevated LDL-cholesterol, hypertension, depression, air pollution, low education, social isolation, cognitive inactivity, hearing and visual loss account for up to 45% of dementia cases.^1^ Further emerging individual-level risk factors include sleep difficulties, unhealthy diet, job strain, and environmental risk factors such as neighborhood deprivation, low financial resources, or lack of access to green spaces.^2^ These findings suggest the potential for dementia risk reduction through risk factor modification.

Owing to the multifactorial etiology of dementia, targeting several risk factors simultaneously constitutes a particularly promising approach. The Finnish Geriatric Study to Prevent Cognitive Impairment and Disability (FINGER) was the first multidomain intervention study reporting benefits of a lifestyle intervention (enhancement of physical, social and cognitive activity, optimization of nutrition, management of cardiovascular risk factors) on cognitive performance and several secondary outcomes in an at-risk population after 24 months.^3^ These findings led to the launch of the World-Wide FINGERS (WW-FINGERS) network, aiming to tailor the approach of FINGER to different regional and socioeconomic contexts.^4^

A particular challenge of lifestyle interventions against cognitive decline and dementia is the choice of suitable and meaningful outcomes. Several other multidomain trials in older at-risk samples reported no intervention effects on cognitive outcomes in the total sample, e.g., the French Multidomain Alzheimer Prevention Trial (MAPT^5^;) or the Dutch Prevention of Dementia Through Intensive Vascular Care (preDIVA^6^;)-trials. Recent trials, however, e.g., the US POINTER-trial and the Korean SUPERBRAIN feasibility trial, reported beneficial effects of multidomain lifestyle interventions on cognitive performance.^7,8^ Overall, meta-analyses report significant but rather small effects of respective interventions on cognitive outcomes.^9^ It has been suggested that lifestyle trials might require longer intervention periods than currently applied (commonly between one and two years) to manifest in changes in cognitive performance. Dementia risk scores, quantifying an individual's risk for dementia/room for improvement due to lifestyle changes have been deemed a promising outcome for intervention trials targeting older at-risk populations, as they are highly responsive to change, e.g., capturing improvements in lifestyle and modifiable risk factors due to an intervention, which might translate to lower risk of cognitive decline and dementia in the long-run.^10^ Due to the potentially long prodromal stages of AD and vascular dementia, neuroimaging markers have been suggested as sensitive measures of intervention effectiveness in (multidomain) lifestyle interventions.^11^

Sufficient evidence exists on the links of single risk factors for dementia^12–18^ or combinations of risk factors^19–21^ with imaging markers of neurodegeneration and small vessel disease, while less is known about possible mechanisms of the effects of (multidomain) lifestyle interventions on neuroimaging outcomes. Regarding randomized controlled trials (RCTs), certain studies found beneficial effects of physical activity interventions on hippocampal volume,^22–24^ while evidence on the effects of dietary interventions is less conclusive.^25–27^ In the FINGER-trial, no intervention effects on neuroimaging markers were detected,^11^ while the pilot study of the South Korean SUPERBRAIN-study, adapting the FINGER-intervention, found positive effects of a facility-based multidomain intervention on cortical thickness and brain-derived neurotrophic factor (BDNF) after a 24-weeks intervention.^28^ A recent review reported mixed findings regarding effects of multidomain interventions on neuroimaging biomarkers.^29^ To the best of our knowledge, no study to date investigated whether intervention effects on dementia risk scores are linked to changes in imaging markers of brain health.

In Germany, the AgeWell.de-trial was the first study to implement a FINGER-like multidomain intervention in older general practitioner (GP) patients at increased risk for dementia, targeting optimization of nutrition and medication, enhancement of physical, cognitive and social activity, management of vascular risk factors and depressive symptoms, if applicable. While no effects on global cognitive performance, assessed using a global composite z-score comprising six domain-specific cognitive tests, was observed^30^ at 24 months follow-up, the intervention improved depressive symptoms in women and successfully reduced lifestyle-based risk for dementia in the total sample, assessed using a validated dementia risk score.^31^ These beneficial effects were driven particularly by improvements in adherence to a healthy diet and blood pressure in the intervention group. Similar findings have been reported for other multidomain intervention trials, using risk scores such as the Lifestyle for Brain Health (LIBRA)-index,^32^ the Cardiovascular Risk Factors, Aging and Dementia (CAIDE)-score^33^ or the Australian National University AD Risk Index (ANU-ADRI).^34^ This provides evidence that multidomain interventions are apt to improve participants’ risk profiles for dementia.^10,35,36^ If these beneficial lifestyle changes are maintained beyond the intervention period, they might contribute to lower rates of incident dementia on a population level in the long run.

As a sub-study of the multi-centric AgeWell.de-trial, participants at the Leipzig study site underwent two magnetic resonance imaging (MRI)-assessments at 3T, assessing hippocampal volume (HCV) and entorhinal cortex thickness (ECT) as markers of medial temporal lobe atrophy, and free water fraction (FW), peak width of skeletonized mean diffusivity (PSMD), white matter hyperintensity (WMH) volume, and mean gray matter cerebral blood flow (CBF) as markers related to vascular pathology. While no intervention effect on neuroimaging markers were detected, results suggested an increase in CBF, which was partly explained by decreased systolic blood pressure, a main effect of the intervention.^37^ In light of the beneficial intervention effects on dementia risk profiles reported earlier,^31^ we aimed to investigate whether changes in dementia risk profiles, assessed using the LIBRA-index, were linked to changes in neuroimaging markers.

## Methods

### Study design and participants

AgeWell.de was a cluster-RCT, conducted at five study sites across Germany (Leipzig, Greifswald, Kiel, Munich, Halle), targeting older (60–77 years) GP patients at increased risk for dementia, according to a CAIDE-score of ≥9 points. The respective cutoff was chosen as it was shown to predict dementia risk with a sensitivity of 0.77 and a specificity of 0.63 in the population-based CAIDE study within a 20-year time period.^33^ GP practices (clusters) were randomized in a 1:1-ratio to either the intervention- or the control group (IG, CG). While IG-participants received a multidomain intervention, i.e., optimization of nutrition and medication, enhancement of physical, social and cognitive activity, management of cardiovascular risk factors, and an intervention targeting depressive symptoms and grief after bereavement (if applicable) for 24 months, CG-participants received GP treatment as usual and general health information on the intervention components. Study design,^38^ baseline participant characteristics^39^ and effects on primary outcomes^30,31^ are reported in detail elsewhere. Baseline assessments (n = 1.030) took place between 06/2018 and 10/2019, follow-up assessments (n = 819) were conducted between 07/2020 and 01/2022. AgeWell.de was prospectively registered in the German Clinical Trials Register (DRKS; trial identifier: DRKS00013555).

Trained study nurses instructed IG-participants on how to conduct the intervention during an in-person visit at participants’ homes, following the baseline assessment. The physical activity component included standardized exercises for strength and balance/flexibility to be conducted at home at least twice a week, and aerobic exercises based on participants’ preferences 3–5 times per week. For optimization of nutrition, participants were instructed to follow the guidelines of the German Nutrition Society, e.g., consumption of ≥5 portions of vegetables and fruit daily, limiting intake of sugar and salt and regular consumption of fish. The cognitive activity component included use of the cognitive training software NeuroNation ^©^ using tablet computers provided by the study team, ≥3 times per week for ≥15 min, respectively. Social activities to be conducted on a regular basis were scheduled individually with participants. If applicable, participants received oral and written information on vascular risk factors (e.g., smoking) and ways to reduce respective risk factors. In case of depressive symptoms, participants were encouraged to contact their attending GP and further received contact information of local self-help groups and helplines to be contacted if necessary. Optimization of medication was based on information on participants’ medication and lab values for hemoglobin A1c and creatinine, provided by attending GPs, and participants’ information on actual current medication. Said information was evaluated to identify potentially inappropriate medication, e.g., anticholinergic drugs, using a list of medications based on Durán and colleagues^40^ and Gray and colleagues,^41^ adapted for the German drug market. Based on this evaluation, standardized letters with recommendations on participants’ medication were sent to attending GPs, including suggestions for modification, if applicable. Study nurses documented adherence to intervention components on nutrition, physical, social, and cognitive activity in regular telephone-based monitoring and booster sessions, after 2, 4, 8, 16, and 20 months, assessing to which degree participants were able to reach their respective goals in the intervention components (response options: not at all (0) –absolutely (4)). Respective values were summed up across intervention components and time points, resulting in a score ranging from 0 to 28.

All AgeWell.de-participants at the Leipzig study site without major MRI exclusion criteria (stents, prosthesis/implants, tattoos/permanent makeup, metal clips, claustrophobia) were offered to participate in the MRI sub-study (n_baseline_: 274). Of 110 interested participants, 56 were included in the study and performed the Mnemonic Similarity Test (MST), and 54 completed the baseline MRI-imaging. At follow-up, 41 participants returned for the second MRI-assessment and performed the MST. Five CG-participants dropped out of the MRI sub-study among whom one refused MRI participation, two declined due to health reasons and two refused due to ongoing Sars-Cov2 pandemic. One discontinued participation in the AgeWell.de-trial. Three IG-participants discontinued the MRI-sub-study because of health reasons (2) and Sars-Cov2-pandemic (1), and four dropped out of the AgeWell.de-trial. Reasons for discontinuing participation in the trial included health-related issues (n = 2, IG), refusal (n = 2, IG) and non-response (n = 1, CG). Full details on the MRI sub-study have been described by Beyer and colleagues.^37^

### Outcomes and covariates

At baseline and follow-up, participants underwent anatomical and functional MRI assessment on a 3 Tesla Siemens MAGNETOM Skyra scanner with a 32-channel head coil at the facilities of the Max Planck Institute for Human Cognitive and Brain Sciences in Leipzig. Assessed imaging markers applied as outcomes in the present study included (head size-corrected) HCV, entorhinal cortex (EC) thickness, WMH volume, free water fraction (FW), peak width of skeletonized mean diffusivity (PSMD), and mean gray matter CBF.

HCV and EC thickness were derived from a MP2RAGE^42^ (TI 1 = 700 ms, TI 2 = 2500 ms, TR = 5000 ms, TE = 2.92 ms, 1 × 1 × 1 mm3) sequence. Noise was removed using MPRAGize and surface reconstruction and segmentation was performed using FreeSurfer version 7.4.1 including the FLAIR image for improved pial placement.^43^ We then ran the Freesurfer longitudinal stream to ensure high within-subject reliability of GM structural measures.^44^ We extracted HCV and estimated total intracranial volume (eICV) and ECT from the Desikan-Killiany parcellation. We averaged HCV and ECT over hemispheres and adjusted HCV for head size according to the following formula: hippocampal volumeadjusted, i = hippocampal volumeraw, i − β * (ICVraw, i − ICVmean).

FW and PSMD were derived from diffusion-weighted imaging acquired with CMRR echo planar imaging (TR 6420 ms, TE 100 ms, FOV 220 × 220 mm2, resolution: 128 × 128, 72 slices, 1.7 × 1.7 × 1.7 mm3, 60 diffusion directions, b = 1000 s/mm2, multiband-factor 2) using the MarkVCID2 FW Biomarker Kits.^45^

WMH volumes were calculated from fluid-attenuated inversion recovery, acquired with 3D turbo spin-echo sequence (TR 5000 ms, TE 395 ms, TI 1800 ms, FOV 230 × 230 mm2, resolution: 192 × 256 × 256, sagittal orientation, 0.9 × 0.9 × 0.9 mm3). We used the SHIVA-WMH detector, a deep-learning-based software tool developed for the automatic detection and segmentation of WMH.^46^ WMH probability was then predicted from T1w and FLAIR images and WMH volumes were extracted as the sum of all voxels with WMH probability >0.5 as recommended and asinh-transformed for statistical analysis.

Arterial spin labeling (ASL) was performed using a pseudo-continuous labeling scheme (pCASL) with a labeling plane 65 mm below the nasal root, applied for 3000 ms using an in-house optimized pulse train.^47^ After a post-labeling delay of 1200 ms, 24 slices were acquired using a gradient-echo EPI readout (matrix: 64 × 64; in-plane resolution: 3 × 3 mm^2^; slice thickness: 4 mm; gap: 0.4 mm; TR/TE: 5020/9.2 ms; GRAPPA factor: 2). The acquisition included 30 label/control pairs and two baseline volumes (total acquisition time: 5:30 min). For anatomical reference, a 3D FLAIR sequence was acquired (TR/TE/TI: 5000/395/1800 ms; resolution: 0.9 × 0.9 × 0.9 mm^3^; GRAPPA factor: 2). All scans were reviewed by study physicians and rated as usable, with no signs of tumors or acute pathology.

CBF was derived as difference between label-control pairs, adjusted for motion and converted to absolute units using a two-compartment model.^48^ We extracted average CBF from high-reliability voxels with gray matter probability >0.9 derived from the T1w image using SPM which were present in both time points. All scans were reviewed by study physicians and rated as usable, with no signs of tumors or acute pathology. A detailed description of MRI procedures, pre-processing and quality control measures undertaken can be found in Beyer and colleagues.^37^

All participants completed the MST^49^ at baseline and follow-up. In this computerized assessment targeting pattern separation performance, a marker of hippocampal integrity, participants were presented 192 colored photographs of objects (publicly available under: https://faculty.sites.uci.edu/starklab/mnemonic-similarity-task-mst/) First, participants indicated whether presented objects belonged inside or outside by pressing a button (128 items total, 2 s object presentation, 0.5 s ISI). Thereafter, 64 of said objects were presented a second time, along with 64 new and 64 similar images (lures), and participants rated the images as “old”, “similar” or “new” by pressing a button (2 s object presentation, 0.5 s ISI, after which the next item was shown with 0.5 s ISI). The lure discrimination index (LDI) was calculated as the difference between the number of lure items rated as “similar” minus new items rated as “similar”. Further, an indicator of recognition memory (REC) was calculated, based on the difference between number of correctly recognized old images minus number of new items identified as old.

Participants’ risk profile for dementia was assessed using the LIBRA-index. LIBRA is based on a systematic review and Delphi consensus study. It has been extensively validated to predict cognitive performance/decline, mild cognitive impairment (MCI), structural changes in neuroimaging markers and incident dementia in population-based cohort studies,^32,50–52^ and has been used as a (surrogate) outcome in lifestyle interventions targeting cognitive decline and dementia risk.^10,31,35^ In its original version, LIBRA comprised 12 modifiable risk (coronary heart disease, diabetes mellitus, hypercholesterolemia, arterial hypertension, depression, obesity, smoking, physical inactivity, chronic kidney disease) and protective factors (low-to-moderate alcohol consumption, high cognitive activity, healthy diet), resulting in a weighted sum score (range: −5.9 to 12.7, higher scores indicating higher risk for dementia). Information on all 12 risk/protective factors included in LIBRA were available in AgeWell.de, based on self-administered questionnaires (healthy diet, alcohol consumption), interviews (depression, smoking, physical inactivity, cognitive activity), anthropometric measures (obesity, hypertension) and information on diagnoses and lab values provided by GPs (hypertension, hypercholesterolemia, diabetes, renal dysfunction, coronary heart disease). Details on operationalization and assessment of each LIBRA-factor is provided in Supplemental Table 1. LIBRA was chosen over other dementia risk scores as it solely comprises modifiable risk factors. While CAIDE also uses information on education and sex, LIBRA was deemed particularly suitable to assess an individual's room for improvement by lifestyle changes for dementia risk reduction.

Participants provided information on sociodemographic characteristics (age, sex, education) during the baseline assessment of the AgeWell.de-trial, conducted by trained study nurses at participants’ homes. Education was assessed using the Comparative Analysis of Social Mobility in Industrial Nations (CASMIN)-classification,^53^ comprising formal and professional education, and grouped into categories low, middle, and high.

### Statistical analyses

Group comparisons between IG and CG were conducted using Chi^2^-tests, t-tests or one-way ANOVA, as appropriate. We assessed associations between lifestyle (LIBRA-score), neuroimaging markers and cognitive performance (LDI, REC) at baseline, controlling for age, sex and education, using multivariable linear regression analyses. Associations of changes in LIBRA-scores with imaging markers at follow-up (EC thickness, HCV, WMHV, PSMD, FW, CBF) were investigated using multivariable linear regression analyses for each imaging marker, controlling for age, sex, education, and baseline-value of respective imaging markers to account for baseline differences between groups and counteract risk of regression to the mean.^54,55^ To assess whether intervention effects on LIBRA-scores predicted changes in imaging markers, interaction terms of changes in lifestyle (LIBRA) with intervention group allocation were included into each model. All regression analyses were run using cluster-robust sandwich estimators to account for clustering of participants in GP practices (n = 23 GP practices; median (IQR) of participants recruited per practice: 2 (1–2)).

To avoid loss of power due to decreased sample size, missing values in components of the LIBRA were imputed using multiple imputations by chained equations,^56^ applying logistic regression with augmentation. Data was missing for the following variables: alcohol consumption at baseline (n = 5), smoking at baseline (n = 1), cognitive activity at baseline (n = 2), alcohol consumption at follow-up (n = 8), smoking at follow-up (n = 2), cognitive activity at follow-up (n = 1), coronary heart disease at follow-up (n = 3), renal dysfunction at follow-up (n = 3), hypertension at follow-up (n = 1), and diabetes at follow-up (n = 3), with missing values imputed as described above. Missing values on LDI and REC at baseline (n = 6, respectively) and follow-up (n = 1, respectively) were not imputed. Likelihood of missing values in the LIBRA-index was unrelated to sex (*p* = 0.364), level of education (*p* = 0.987) or age (*p* = 0.539), therefore, no indication of systematic missingness was detected. We generated 20 imputed datasets with 300 iterations per dataset, following a burn-in of 5 iterations to maximize stability of the imputation algorithm and applying a random seed to allow reproducibility. Participants’ age (no missing values) was registered as a predictor to guide the imputation process. The LIBRA-index at baseline and follow-up was calculated following the imputation. All regression results are based on the results of n = 20 pooled datasets. Missing values on any MRI-markers were not imputed. The alpha-level was set at 0.05 (two-tailed) in all analyses. For multivariable regression analyses, education levels low (n = 1) and middle (n = 22) were grouped together to facilitate interpretation and maximize statistical power. Analyses were conducted using Stata 16.0 (StataCorp).

## Results

### Descriptive analyses

Of 56 individuals who participated in the baseline MRI-assessment (M _age_ (SD): 68.8 (4.2); IG/CG: 24/32; men/women: 30/26), 41 completed the follow-up MRI-assessment after an average of 28 months (IG/CG: 16/25, men/women: 22/19).

Table 1 describes baseline characteristics of the study sample (unimputed data). While IG-participants were slightly older and less likely to be female, these differences were not significant. IG- and CG-participants differed regarding level of education (*p* = 0.010), and IG-participants more often had a diagnosis of hypertension (*p* = 0.005). No further group differences were detected. Adherence to the intervention components (IG only) ranged from 10 to 28 points, higher values indicating better adherence.

**Table 1.** Characteristics of study sample at baseline.

	**Total (n** **=** **41)**	**Intervention group (n** **=** **16)**	**Control group (n** **=** **25)**	**p**
*Sociodemographic information*				
Age, M (SD)	68.1 (4.1)	69.4 (4.0)	67.3 (4.0)	0.099
Female, % (n)	46.3 (19)	31.3 (5)	56.0 (14)	0.121
Education, % (n)				**0**.**010**
Low	2.4 (1)	6.3 (1)	0	
Middle	53.7 (22)	25.0 (4)	72.0 (18)	
High	43.9 (18)	68.8 (11)	28.0 (7)	
*LIBRA-factors*				
Hypertension*, % (n)*	61.0 (25)	87.5 (14)	44.0 (11)	**0**.**005**
Hypercholesterolemia*, % (n)*	53.7 (22)	37.5 (6)	64.0 (16)	0.097
Obesity*, % (n)*	26.8 (11)	37.5 (6)	20.0 (5)	0.217
Physical inactivity*, % (n)*	63.4 (26)	68.8 (11)	60 (15)	0.570
Diabetes*, % (n)*	68.3 (28)	56.3 (9)	76.0 (19)	0.185
Renal dysfunction*, % (n)*	14.6 (6)	6.3 (1)	20.0 (5)	0.224
Coronary heart disease*, % (n)*	7.3 (3)	6.3 (1)	8.0 (2)	0.834
Depression*, % (n)*	9.8 (4)	0	16.0 (4)	0.092
Smoking*, % (n)*	7.5 (3)	12.5 (2)	4.2 (1)	0.327
Low/moderate alcohol consumption*, % (n)*	8.3 (3)	0	14.3 (3)	0.126
Healthy diet*, % (n)*	56.1 (23)	37.5 (6)	68.0 (17)	0.055
High cognitive activity*, % (n)*	41.0 (16)	46.7 (7)	37.5 (9)	0.571
LIBRA-index, total; M (SD)	1.8 (2.5)	2.1 (2.3)	1.7 (2.6)	0.603
Intervention adherence, total; M (SD)		21.9 (5.4)	n.a.	n.a.
*Imaging markers*				
EC thickness (mm; M (SD))	3.0 (0.2)	3.0 (0.2)	3.0 (0.2)	0.834
HCV (mm^3^; M (SD))	3355 (278)	3276 (244)	3406 (294)	0.148
WMH volume (cm^3^; M (SD))	3814 (4720)	3401 (3879)	4078 (5248)	0.660
PSMD (mm^2^/s; M (SD))	3.6*10^−4^ (0.6*10^−4^)	3.7*10^−4^ (0.6*10^−4^)	3.5*10^−4^ (0.5*10^−4^)	0.168
FW fraction, M (SD)	0.22 (0.03)	0.22 (0.02)	0.21 (0.03)	0.562
CBF, M (SD)	53.1 (23.5)	46.6 (11.1)	57.2 (28.3)	0.160
LDI, M (SD)	0.2 (0.2)	0.1 (0.1)	0.2 (0.2)	0.383
REC, M (SD)	0.8 (0.1)	0.8 (0.1)	0.8 (0.2)	0.989

CBF: cerebral blood flow; EC: entorhinal cortex; FQ: free water; HCV: hippocampal volume; LIBRA: lifestyle for brain health; LDI: Lure Discrimination Index from Mnemonic Similarity Test (MST); M: mean; PSMD: peak width of skeletonized mean diffusivity; REC: Recognition score from MST; SD: standard deviation; WMH: white matter hyperintensities.

Participants in the AgeWell.de imaging study did not differ from the total AgeWell.de-sample (n = 1030) at baseline regarding age (*p* = 0.272), sex (*p* = 0.448), or global cognitive performance (*p* = 0.184), but more often had a high level of education (*p* < 0.001) and lower LIBRA-scores (*p* = 0.040) than participants in total sample, indicating lower modifiable dementia risk (not tabulated).

At baseline, lower LIBRA-scores, indicating a lower risk for dementia, were linked to larger HCV (b = -0.37.59, 95% CI: −64.06; −11.12; 0.001; *p* = 0.008), controlling for age, sex and level of education. No associations between further imaging markers, cognitive performance (LDI, REC) and LIBRA at baseline were detected (see Supplemental Table 2).

### Effects of lifestyle changes on neuroimaging markers

Overall, LIBRA-scores decreased both in IG- and CG-participants (mean change _IG_: −0.37, 95% CI: −0.67, −0.07; mean change _CG_: −0.06, 95% CI: −0.28, 0.17), however, change in LIBRA-scores between baseline and follow-up did not differ between groups (ANOVA, Chi-square: 0.0237, *p* = 0.086; not tabulated).

Effects of changes in lifestyle (LIBRA) on MRI-markers are described in Table 2. No effects of lifestyle changes on *EC thickness* were detected (b = 0.007; 95% CI: −0.004; 0.02), the interaction of LIBRA and group allocation marginally missed significance (b = -0.02; 95% CI: −0.05; 0.0008; *p* = 0.051).

**Table 2.** Multivariable regression analyses assessing effects of lifestyle changes on imaging markers at follow-up.

Outcome	Predictors	b	95% CI	p
**EC thickness**				
	Intervention group (ref.: control group)	−0.08	−0.18; 0.02	0.097
	Δ LIBRA	0.01	0.00; 0.02	0.183
	Δ LIBRA*intervention group	−0.02	−0.05; 0.00	0.057
**HCV**				
	Intervention group (ref.: control group)	1.80	−52.24; 55.85	0.945
	Δ LIBRA	7.73	1.20; 14.26	**0**.**023**
	Δ LIBRA*intervention group	−13.49	−32.97; 5.99	0.164
**WMH volume**				
	Intervention group (ref.: control group)	447.46	−195.60; 1090.53	0.162
	Δ LIBRA	69.77	−53.09; 192.62	0.246
	Δ LIBRA*intervention group	5.28	−162.02; 172.58	0.948
**PSMD**				
	Intervention group (ref.: control group)	.0.00001	−0.00003; 2.16*10^−6^	0.087
	Δ LIBRA	−6.87*10^−7^	−8.08*10^−6^; 3.71*10^−6^	0.748
	Δ LIBRA*intervention group	−1.36*10^−6^	−7.53*10^−6^; 4.80*10^−6^	0.649
**FW fraction**				
	Intervention group (ref.: control group)	0.01	0.00; 0.02	0.135
	Δ LIBRA	0.0003	−0.002; 0.003	0.869
	Δ LIBRA*intervention group	−0.0006	−0.004; 0.003	0.752
**CBF**				
	Intervention group (ref.: control group)	4.97	−1.75; 11.70	0.139
	Δ LIBRA	−1.27	−2.92; 0.38	0.125
	Δ LIBRA*intervention group	1.48	−0.68; 3.63	0.168

Multivariable linear regression, outcomes: MRI-imaging markers at follow-up, using n = 20 imputed datasets; all analyses controlling for baseline values of respective outcome, age, sex, and education (assessed using the Comparative Analysis of Social Mobility in Industrial Nations (CASMIN)-scale). CBF: cerebral blood flow; CI: confidence interval; ECT: entorhinal cortex thickness; FW: free water; HCV: hippocampus volume; PSMD: peak width of skeletonized mean diffusivity; WMH: white matter hyperintensity.

**Figure 1.**
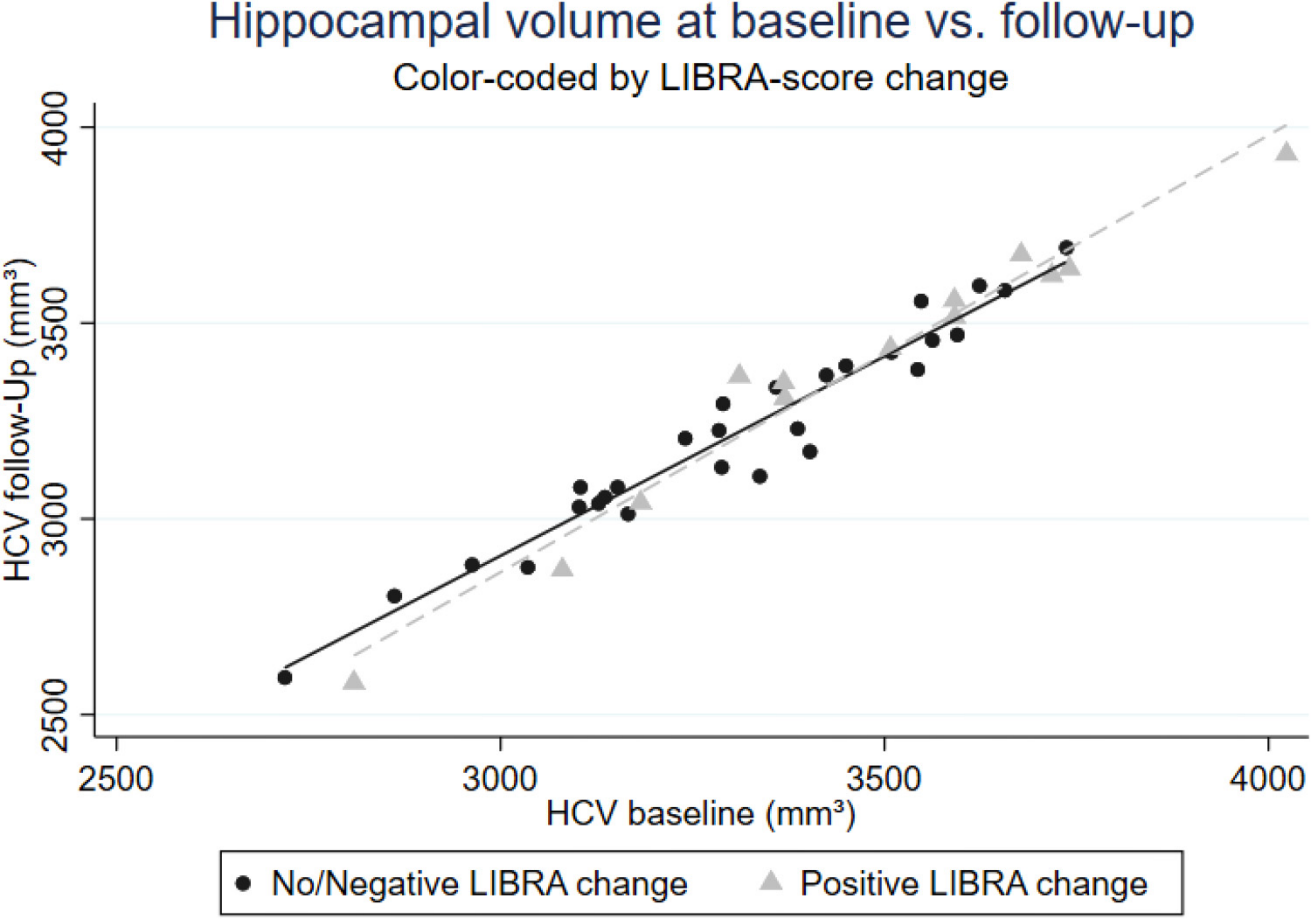
Hippocampal volume at baseline and follow-up by change in LIBRA.

An increase in LIBRA (i.e., detrimental lifestyle changes) was significantly associated with a smaller decline in *HCV* (b = 7.73; 95% CI: 1.20; 14.26; *p* = 0.023). Hippocampal volume at baseline and follow-up by change in LIBRA (positive vs. negative/no change) is displayed in Figure 1). The interaction between group allocation and change in LIBRA was negative but did not reach statistical significance (b = −13.49; 95% CI: −32.97; 5.99). Neither changes in lifestyle, nor group allocation or the interaction between group allocation and lifestyle changes was linked to changes in *WMH volume, PSMD, FW fraction, or CBF*.

Further exploratory analyses assessed whether changes in lifestyle were linked to changes on cognitive performance (*LDI, REC*). Changes in *REC* were not linked to changes in LIBRA-scores (b = 0.004, 95% CI:-0.01; 0.02), group allocation (b = 0.01; 95% CI: −0.09; 0.11) or the interaction of LIBRA and group allocation (b = -0.02; 95% CI: −0.05; 0.006). Lifestyle changes (b = 0.01; 95% CI: −0.01; 0.04) or intervention group allocation (b = -0.13; 95% CI: −0.27; 0.00) were not linked to changes in LDI-performance. However, in the intervention group, (detrimental) changes in lifestyle were linked to decreased LDI-performance, as indicated by the interaction term (b = -0.04; 95% CI: −0.08; −0.004; *p* = 0.034; not tabulated).

To assess whether changes in cognitive performance were linked to changes in imaging markers, multivariable regression models for each imaging marker were run, using ΔLDI (LDI_FU_ - LDI_BL_) and ΔREC (REC_FU_ - REC_BL_) as predictors, controlling for age, sex, education and baseline values of imaging markers, respectively. Greater changes in REC were linked to greater EC thickness at follow-up (b = 0.27, 95% CI: 0.10, 0.44; *p* = 0.004). No further associations between ΔLDI or ΔREC were detected (see Supplemental Table 3).

Sensitivity analyses within IG-participants (n = 16) assessed potential influence of intervention adherence (total score) on imaging markers and cognitive performance, controlling for changes in lifestyle (ΔLIBRA), age, sex, education, and baseline values of respective outcomes. Better adherence to the intervention was not linked to changes in neuroimaging markers or cognitive performance (all *p* ≥ 0.099; see Supplemental Table 4).

Changes in individual factors of LIBRA in IG- and CG-participants are described in Supplemental Table 5. Overall, no changes in the risk factors hypercholesterolemia, coronary heart disease or smoking were observed during the intervention period. IG participants more often improved their risk profile for dementia regarding hypertension (*p* = 0.017), physical inactivity (*p* = 0.017) and consumption of a healthy diet (*p* = 0.020). However, the number of IG participants exhibiting the risk factors depression and diabetes increased slightly during the intervention period, although between-group differences were non-significant (*p* = 0.246 and 0.636, respectively). Change in the other LIBRA-factors did not differ by group allocation.

Improvements in the risk factors obesity (b = 0.12; 95% CI: 0.03; 0.21) and alcohol consumption (b = 0.19; 95% CI: 0.08; 0.31) were linked to higher EC thickness at follow-up, but both unrelated to group allocation, when controlling for covariates (Supplemental Table 6). Improvements in depression were associated with lower EC thickness (b = -0.13; 95% CI: −0.23; −0.03), while this risk factor changed only in CG participants. Participants who improved in the risk factor obesity had lower HCV at follow-up (b = -112.61; 95% CI: −156.84; −68.38), which was however not linked to the intervention. Participants who improved the risk factor renal dysfunction (CG only, n = 2) had larger HCV at follow-up (b = 58.56; 95% CI: 8.00; 109.11). Higher CBF at follow-up was observed in participants who improved the risk factor hypertension (b = 8.89; 95% CI: 0.13; 17.64), but independent of the intervention. Lower CBF was found in those who improved regarding depression (b = -17.23; 95% CI: −30.48; −3.98), but unrelated to group allocation.

## Discussion

This study aimed to describe whether changes in dementia risk profiles, assessed using a validated risk score (LIBRA), were linked to structural brain changes and vascular markers in a sub-sample of the German AgeWell.de-trial. While participants’ risk profiles for dementia improved in both groups in the present sub-study, these effects did not induce changes in EC thickness, HCV, WMH, FW, PSMD or CBF. Detrimental lifestyle changes were linked to smaller reductions in hippocampal volume, however, this association was not related to the intervention.

Several studies have reported cross-sectional associations between structural brain health markers or white matter connectivity and LIBRA-scores in middle-aged and older adults.^52,57,58^ Aligning with these findings, lower LIBRA-scores, indicating a lower risk for dementia, were linked to larger HCV at baseline in our study. While certain cohort studies found no longitudinal association between LIBRA and WMH volume,^58^ others detected associations between dementia risk scores, e.g., CAIDE, and brain atrophy at follow-up.^59^ In the FINGER-trial, higher values on the cardiovascular risk scores of the European Society of Cardiology (SCORE, SCORE-OP) were cross-sectionally linked to lower HCV, total GMV, cortical thickness, and higher WMH volume, but no associations between changes in SCORE/SCORE-OP and imaging markers were detected.^60^ No intervention effects on regional brain volumes, cortical thickness and white matter lesions were detected in FINGER; however, greater intervention benefits on processing speed were detected in participants with higher cortical thickness at baseline.^11^ On the other hand, FINGER reported less accumulation of amyloid beta in intervention group participants with a higher genetic risk for AD.^61^ Reductions in CAIDE were linked to less decline in hippocampal volumes in intervention group participants of FINGER.^62^ In the recently completed US-POINTER-trial, testing a FINGER-like multidomain intervention in the US, higher values on the Framingham Risk Score at baseline were associated with lower hippocampal and whole cerebral GM volume, fractional anisotropy in WM and GM CBF, as well as higher WMH volume and FW.^63^

Regarding dementia risk scores, several multidomain interventions reported beneficial intervention effects similar to those shown in the complete AgeWell.de sample^31^ and marginally significant in the MRI sub-sample. For example, the FINGER-intervention improved participants’ LIBRA-scores,^35^ and beneficial intervention effects on CAIDE and LIBRA were found for the MAPT-, preDIVA- and Healthy Aging Through Internet Counselling in the Elderly (HATICE)-trials.^10^ Recently, the 3-year Maintain Your Brain-trial, which assessed effects of a remote multidomain lifestyle intervention, reported beneficial effects on the ANU-ADRI-score.^36^ However, these trials did not assess whether effects on dementia risk scores correspond to changes in neuroimaging markers.

Overall, our results suggest no association between beneficial changes in a validated dementia risk score and structural brain health markers. Certain studies suggest that respective lifestyle changes might require more time to manifest in measurable improvements in imaging markers, as most (multidomain) lifestyle interventions currently employ intervention periods of one or two years.^61^ What is more important, AgeWell.de was designed as a pragmatic intervention with a focus on feasible implementation in real-world settings, therefore, the intervention was conducted independently by participants, supported by regular telephone-based contacts with study nurses. Possibly, more intensive interventions might be necessary to result in observable changes in neuroimaging markers. In line with this, the South-Korean SUPERBRAIN pilot trial reported beneficial intervention effects on cortical thickness and BDNF after an intensive 24 weeks multidomain intervention, carried out at the study facilities, while no respective findings were observed in the home-based intervention group.^28^ Evidence from a cohort of older adults with a family history of AD (PREVENT AD) suggests that between-group differences in functional connectivity and GM markers were particularly pronounced between individuals who adhered to several features of a healthy lifestyle simultaneously and those who were non-adherent.^19^ In the total AgeWell.de-sample, on the other hand, only certain components of the LIBRA were amendable by the intervention, i.e., diet, hypertension, and cognitive activity (the latter in younger participants only^31^), while improvements in hypertension, physical inactivity and nutrition were observed in IG-participants in the present subsample. This further suggests that higher-intensity/more tailored interventions might be necessary to induce even greater changes in dementia risk profiles and corresponding changes in neuroimaging markers.

Validated risk scores like LIBRA could be used in clinical or community settings to identify individuals with modifiable risk factors and to communicate personalized risk profiles, which has been shown to be feasible in a recent study from the Netherlands.^64^ Respective individualized feedback might aid in visualizing the potential impact of lifestyle changes and increase motivation to adhere to multidomain interventions. Tailoring intervention content and intensity based on modifiable lifestyle risk might enhance efficacy and sustainability of lifestyle changes in at-risk populations. Furthermore, we enrolled a high-risk sample for cognitive decline and AD (CAIDE-score ≥ 9 points), which might have affected impact of the intervention. Still, it has to be noted that the present study employed a small sample size, limiting potential for conclusive statements on the role of lifestyle changes on imaging markers.

The positive association between increases in LIBRA score and a smaller reduction in HCV was counterintuitive. One possible explanation is the association between higher HCV and lower LIBRA-scores at baseline, indicating less room for improvement and resulting in greater absolute volume loss over time. (regression to the mean or ceiling effects). What is more, participants in the MRI subsample exhibited slightly higher average LIBRA scores at baseline than the overall AgeWell.de-sample (M (SD): 1.8 (2.5) versus 1.5 (2.7)). This may reflect selective participation of individuals with greater health concerns or known risk factors, potentially attenuating or distorting expected associations. Further studies applying larger samples are needed to clarify these trends. Analyses of single LIBRA factors indicated that only certain risk factors, i.e., hypertension, physical inactivity, and diet, improved in the IG, whereas other components worsened slightly during the same period. This might indicate that the overall LIBRA does not adequately reflect beneficial behavior changes that are important for hippocampal integrity in our high-risk sample of older adults. Further associations of changes in single risk factors were detected, including a beneficial effect of improvement in the risk factor hypertension on CBF at follow-up. However, the respective associations were independent of group allocation and driven by small numbers of individuals showing changes in the individual risk factors. Therefore, these findings should be interpreted with caution.

In exploratory analyses, a significant group*LIBRA-interaction was detected, indicating that beneficial lifestyle changes were linked to better cognitive performance in the LDI in the IG. Note, however, that changes in LDI as well as overall sample size were rather small, therefore, these results should be interpreted cautiously. No respective associations between lifestyle changes and REC-performance were found.

### Strengths and limitations

Limitations arise from the small sample size, which was likely due to rigorous exclusion criteria for MRI-assessments. Since AgeWell.de targeted a high-risk sample of older adults at increased risk for dementia (inclusion criterion: CAIDE-score ≥ 9 points, assessing conditions like diabetes mellitus or hypertension, increasing probability of implants/stents), this was expected to some degree. Conduct of the follow-up MRI assessment was challenged by the COVID-19 pandemic, as several participants did not want to take the risk of a face-to-face appointment, which may have induced loss to follow-up and a slight time gap (about 4 months) between end of intervention and follow-up MRI assessments. This may have resulted in an insufficient sample size to detect meaningful associations. Due to budgetary constraints, no biomarkers were collected, which could have provided information on the influence of, e.g., *APOE* genotype on the association between lifestyle-based risk factors and neuroimaging markers.

Notable strengths of our study include the cluster-randomized study design, allowing for statements on intervention effects. Further, we applied state-of-the-art imaging techniques assessing markers of atrophy and cerebral small vessel disease, as well as an advanced ASL-protocol to enhance precision of CBF assessments. Statistical analyses followed detailed protocols to strengthen validity of findings, including handling of missing values applying appropriate imputation techniques. This study was conducted in a highly relevant population for health promotion and prevention, i.e., older adults at increased risk for dementia, and contributes to the growing research field on suitable outcomes for intervention trials targeting cognitive decline and dementia risk reduction.^10^ Lastly, information on all n = 12 risk/protective factors comprised by the LIBRA-index were available in our study, maximizing its predictive value.

### Conclusion

The present study investigated whether beneficial changes in dementia risk scores, i.e., the Lifestyle for Brain Health (LIBRA)-index, translate to changes in neuroimaging markers in participants of a two-year multidomain lifestyle intervention. While (detrimental) lifestyle changes were linked to lower levels of hippocampal volume loss, this effect was independent of the multidomain intervention. Analyses of individual risk- and protective factors indicated that only certain factors—i.e., hypertension, physical inactivity, and a healthy diet—improved in IG participants, whereas other components remained unchanged or worsened slightly. No further associations between markers of neurodegeneration and cerebral small vessel disease and intervention effects on dementia risk profiles at follow-up were found. Explanations include the rather small sample size, implying limited power of our analyses, intervention intensity and a comparatively short intervention period. Dementia risk scores constitute promising outcomes for lifestyle interventions targeting brain health and can further be used to monitor intervention adherence. Mounting evidence, including own findings, suggest that lifestyle interventions can improve dementia risk profiles. To date, however, our knowledge regarding mechanistic pathways of these intervention effects is limited. Further studies assessing lifestyle-based dementia risk and corresponding changes in neuroimaging markers, applying larger sample sizes and possibly longer intervention periods, are required to enhance our understanding on underlying mechanisms of the effects of lifestyle interventions in older adults at increased risk for dementia.

## Supplemental Material

sj-docx-1-alz-10.1177_13872877251414423 - Supplemental material for Evaluating MRI correlates of lifestyle-based dementia risk reduction: Results from the AgeWell.de imaging study Supplemental material, sj-docx-1-alz-10.1177_13872877251414423 for Evaluating MRI correlates of lifestyle-based dementia risk reduction: Results from the AgeWell.de imaging study by Andrea E. Zülke, Frauke Beyer, Melanie Luppa, Toralf Mildner, Thomas Frese, Jochen Gensichen, Hanna Kaduszkiewicz, David Czock, Hans-Helmut König, Birgitt Wiese, Wolfgang Hoffmann, Jochen René Thyrian, Arno Villringer, Steffi G. Riedel-Heller and A. Veronica Witte in Journal of Alzheimer's Disease
