# Supplementary material for: Evaluating MRI correlates of lifestyle-based dementia risk reduction: Results from the AgeWell.de imaging study
Source: J Alzheimers Dis. 2026 Jan 13;110(1):258–70. doi: 10.1177/13872877251414423 (PMC12960767; doi:10.1177/13872877251414423)

**Supplemental Material**

**Evaluating MRI correlates of lifestyle-based dementia risk reduction: Results from the AgeWell.de imaging study**

**Supplemental Table 1.** Operationalization and assessment of LIBRA-factors in AgeWell.de

|  |  | Original LIBRA-score | Assessment |
| --- | --- | --- | --- |
|  | **Points** | **Definition** |  |
| Hypertension | **0** | SBP <140 and DBP <90 and no diagnosis of hypertension | Anthropometric examination, conducted by study nurse |
|  | **1.6** | SBP ≥140 and/or DBP ≥90 and/or diagnosed hypertension |  |
| Hypercholesterolemia | **0** | Total cholesterol <6.5 mmol/l and no diagnosed hyperlipidemia/hypercholesterolemia | Lab values provided by attending GP |
|  | **1.4** | Total cholesterol ≥6.5 mmol/l and/or diagnosed hyperlipidemia or hypercholesterolemia |  |
| Obesity | **0** | <30 | Anthropometric examination, conducted by study nurse |
|  | **1.6** | ≥30 |  |
| Physical inactivity | **0** | Moderate or high-intensity physical activity at least 2 times/week for ≥30 minutes, respectively, assessed by PAQ | Assessed during BL/FU-interview |
|  | **1.1** | Not engaging in moderate or high-intensity physical activity at least 2 times/week for ≥30 minutes, respectively, assessed by PAQ |  |
| Diabetes | **0** | No current diagnosis of diabetes mellitus | Diagnosis and lab values provided by attending GP |
|  | **1.3** | Current diagnosis of diabetes mellitus or HbA1c ≥ 6.5% |  |
| Renal dysfunction | **0** | No history of renal dysfunction | Diagnosis provided by attending GP |
|  | **1.1** | History of renal dysfunction |  |
| Coronary heart disease | **0** | No history of coronary heart disease, myocardial infarction, heart failure, peripheral vascular disease, myocardial infarction | Diagnosis provided by attending GP |
|  | **1.0** | History of coronary heart disease, myocardial infarction, heart failure, peripheral vascular disease, myocardial infarction |  |
| Depression | **0** | GDS-15 <6 and/or no diagnosis of depression | Assessed during BL/FU-interview; diagnosis provided by attending GP |
|  | **2.1** | GDS-15 ≥6 and/or diagnosis of depression |  |
| Smoking | **0** | Not current smoker | Assessed during BL/FU-interview |
|  | **1.5** | Current smoker |  |
| Low/moderate alcohol consumption | **-1** | 1-13 units of alcohol/week | Assessed during BL/FU-interview |
|  | **0** | No drinking or ≥ 14 units of alcohol/week |  |
| Healthy diet | **-1.7** | Consumption of ≥ 5 servings of fruit and vegetables/daily or consumption of ≥ 650gr of fruit and vegetables/daily, assessed by FFQ | Assessed during BL/FU-interview |
|  | **0** | Consumption of < 5 servings of fruit and vegetables/daily or consumption of < 650gr of fruit and vegetables/daily, assessed by FFQ |  |
| High cognitive activity | **-3.2** | Highest 30% of cognitive activity score (observed range: 3-71), assessed by CAQ | Assessed during BL/FU-interview |
|  | **0** | Lowest 70% of cognitive activity score (observed range: 3-71), assessed by CAQ |  |

BL: baseline; BMI: body mass index; CAQ: cognitive activities questionnaire;^1^ DBP: diastolic blood pressure; FFQ: food frequency questionnaire;^2^ FU: follow-up; GP: general practitioner; GDS: geriatric depression scale;^3^ LIBRA: lifestyle for brain health; PAQ: physical activities questionnaire;^1^ SBP: systolic blood pressure

**References**

1. Luck T, Riedel-Heller SG, Kaduszkiewicz H, et al. Mild cognitive impairment in general practice: age-specific prevalence and correlate results from the German study on ageing, cognition and dementia in primary care patients (AgeCoDe). *Dement Geriatr Cogn Disord* 2007; 24: 307–316.

2. Haftenberger M, Heuer T, Heidemann C, et al. Relative validation of a food frequency questionnaire for national health and nutrition monitoring. *Nutr J* 2010; 9: 36.

3. Yesavage JA and Sheikh JI. 9/Geriatric Depression Scale (GDS). *Clin Gerontol* 1986; 5: 165–173.

**Supplemental Table 2.** Multivariable regression analyses assessing associations of lifestyle with imaging markers and cognitive performance at baseline

| Outcome | Predictors | b | 95% CI | p |  |
| --- | --- | --- | --- | --- | --- |
| *Neuroimaging markers* | | | | | |
| EC thickness | | | | | |
|  | LIBRA | 0.0003 | -0.02; 0.02 | 0.975 |  |
|  | Female sex (ref.: male) | -0.01 | -0.12; 0.10 | 0.818 |  |
|  | Age | -0.01 | -0.02; -0.01 | **<0.001** |  |
|  | High education (ref.: middle/low) | -0.05 | -0.15; 0.05 | 0.291 |  |
| HCV | | | | | |
|  | LIBRA | -37.59 | -64.06; -11.12 | **0.008** |  |
|  | Female sex (ref.: male) | 34.88 | -76.95; 146.70 | 0.523 |  |
|  | Age | -41.75 | -51.45; -32.04 | **<0.001** |  |
|  | High education (ref.: middle/low) | 35.88 | -100.13; 171.89 | 0.588 |  |
| WMH volume | | | | | |
|  | LIBRA | -104.23 | -494.48; 286.02 | 0.583 |  |
|  | Female sex (ref.: male) | -1530.83 | -5375.10; 2313.45 | 0.416 |  |
|  | Age | 444.25 | 142.66; 745.85 | **0.006** |  |
|  | High education (ref.: middle/low) | -283.17 | -3834.90; 3268.56 | 0.870 |  |
| PSMD | | | | | |
|  | LIBRA | 1.27*10^-6^ | -4.35*10^-6^; 6.78*10^-6^ | 0.637 |  |
|  | Female sex (ref.: male) | -0.00006 | -0.00009; -0.00001 | **0.005** |  |
|  | Age | 3.84*10^-6^ | 7.98*10^-7^; 6.89*10^-6^ | **0.016** |  |
|  | High education (ref.: middle/low) | 5.97*10^-6^ | -0.00003; 0.00004 | 0.727 |  |
| FW fraction | | | | | |
|  | LIBRA | 0.002 | -0.001; 0.004 | 0.249 |  |
|  | Female sex (ref.: male) | -0.01 | -0.03; 0.004 | 0.133 |  |
|  | Age | 0.003 | 0.002; 0.005 | **<0.001** |  |
|  | High education (ref.: middle/low) | 0.005 | -0.01; 0.02 | 0.622 |  |
| CBF | | | | | |
|  | LIBRA | -1.61 | -4.93; 1.71 | 0.324 |  |
|  | Female sex (ref.: male) | 0.85 | -12.50; 14.20 | 0.896 |  |
|  | Age | -0.07 | -1.12; 0.99 | 0.893 |  |
|  | High education (ref.: middle/low) | 2.71 | -8.63; 14.05 | 0.624 |  |
| *Cognitive performance* | | | | | |
| LDI | | | | | |
|  | LIBRA | -0.005 | -0.03; 0.02 | 0.701 |  |
|  | Female sex (ref.: male) | -0.02 | -0.13; 0.09 | 0.687 |  |
|  | Age | -0.005 | -0.02; 0.01 | 0.352 |  |
|  | High education (ref.: middle/low) | -0.002 | -0.23; 1.30 | 0.981 |  |
| REC | | | | | |
|  | LIBRA | -0.05 | -0.03; 0.02 | 0.708 |  |
|  | Female sex (ref.: male) | -0.02 | -0.14; 0.09 | 0.687 |  |
|  | Age | -0.005 | -0.02; 0.007 | 0.393 |  |
|  | High education (ref.: middle/low) | 0.04 | -0.05; 0.13 | 0.327 |  |

Multivariable linear regression, outcomes: MRI-imaging markers at baseline, using n = 20 imputed datasets; education assessed using the Comparative Analysis of Social Mobility in Industrial Nations (CASMIN)-scale. CBF: cerebral blood flow; CI: confidence interval; ECT: entorhinal cortex thickness; FW: free water; HCV: hippocampus volume; LDI: lure discrimination index; PSMD: peak width of skeletonized mean diffusivity; REC: recognition memory; WMH: white matter hyperintensity.

**Supplemental Table 3.** Multivariable regression analyses assessing effects of changes in cognitive performance on imaging markers at follow-up

| Outcome | Predictors | b | 95% CI | p | |
| --- | --- | --- | --- | --- | --- |
| *Neuroimaging markers* | | | | |  |
| EC thickness | | | | |  |
|  | EC thickness at baseline | 0.70 | 0.54; 0.86 | **<0.001** | |
|  | LIBRA at baseline | 0.001 | -0.01; 0.01 | 0.816 | |
|  | Intervention group (ref.: control group) | -0.05 | -0.11; 0.01 | 0.094 | |
|  | ∆LDI | 0.03 | -0.20; 0.26 | 0.773 | |
|  | ∆REC | 0.27 | 0.10; 0.44 | 0.004 | |
| HCV | | | | |  |
|  | HCV at baseline | 1.06 | 0.97; 1.14 | **<0.001** | |
|  | LIBRA at baseline | 3.16 | -8.74; 15.05 | 0.584 | |
|  | Intervention group (ref.: control group) | -0.26 | -46.67; 46.15 | 0.991 | |
|  | ∆LDI | -5.53 | -179.03; 167.96 | 0.947 | |
|  | ∆REC | 144.29 | -126.59; 415.18 | 0.278 | |
| WMH volume | | | | |  |
|  | WMHV at baseline | 1.28 | 1.18; 1.37 | **<0.001** | |
|  | LIBRA at baseline | -78.40 | -179.24; 22.43 | 0.120 | |
|  | Intervention group (ref.: control group) | 600.38 | -126.50; 1327.25 | 0.100 | |
|  | ∆LDI | -845.13 | -2039.54; 331.27 | 0.148 | |
|  | ∆REC | -760.31 | -2482.58; 961.96 | 0.366 | |
| PSMD | | | | |  |
|  | PSMD at baseline | 0.92 | 0.75; 1.08 | **<0.001** | |
|  | LIBRA at baseline | 1.37*10^8^ | -3.66*10^6^; 3.69*10^6^ | 0.994 | |
|  | Intervention group (ref.: control group) | -3.92*10^6^ | -0.00002; 0.00001 | 0.589 | |
|  | ∆LDI | 3.38*10^6^ | -0.00004; 0.00005 | 0.884 | |
|  | ∆REC | -6.47*10^6^ | -0.00007; 0.00005 | 0.833 | |
| FW fraction | | | | |  |
|  | FW at baseline | 1.01 | 0.80; 1.22 | **<0.001** | |
|  | LIBRA at baseline | -0.0002 | -0.003; 0.002 | 0.873 | |
|  | Intervention group (ref.: control group) | 0.009 | -0.005; 0.02 | 0.202 | |
|  | ∆LDI | -0.002 | -0.03; 0.03 | 0.887 | |
|  | ∆REC | -0.002 | -0.05; 0.05 | 0.948 | |
| CBF | | | | |  |
|  | CBF at baseline | 0.03 | -0.13; 0.19 | 0.677 | |
|  | LIBRA at baseline | 0.96 | -1.29; 3.20 | 0.383 | |
|  | Intervention group (ref.: control group) | 1.02 | -8.83; 10.87 | 0.831 | |
|  | ∆LDI | 7.20 | -7.92; 22.32 | 0.330 | |
|  | ∆REC | 11.41 | -10.89; 33.71 | 0.297 | |

Multivariable linear regression, outcomes: MRI-imaging markers at follow-up, using n = 20 imputed datasets. All analyses controlling for baseline values of respective outcome, age, sex, and education (assessed using the Comparative Analysis of Social Mobility in Industrial Nations (CASMIN)-scale). CBF: cerebral blood flow; CI: confidence interval; ECT: entorhinal cortex thickness; FW: free water; HCV: hippocampus volume; LDI: lure discrimination index; PSMD: peak width of skeletonized mean diffusivity; REC: recognition memory; WMH: white matter hyperintensity.

**Supplemental Table 4.** Multivariable regression analyses assessing effects of lifestyle changes and intervention adherence on imaging markers and cognitive performance at follow-up (IG only; n = 16)

| Outcome | Predictors | b | 95% CI | p |  |
| --- | --- | --- | --- | --- | --- |
| *Neuroimaging markers* | | | | | |
| EC thickness | | | | | |
|  | EC thickness at baseline | 0.62 | 0.12; 1.12 | **0.023** |  |
|  | ∆LIBRA | -0.01 | -0.03; 0.001 | 0.065 |  |
|  | Intervention adherence (total score) | -0.0002 | -0.02; 0.02 | 0.978 |  |
| HCV | | | | | |
|  | HCV at baseline | 1.10 | 0.96; 1.24 | **<0.001** |  |
|  | ∆LIBRA | -0.10 | -23.44; 23.23 | 0.922 |  |
|  | Intervention adherence (total score) | 1.40 | -8.66; 11.46 | 0.749 |  |
| WMH volume | | | | | |
|  | WMHV at baseline | 1.30 | 1.20; 1.40 | **<0.001** |  |
|  | ∆LIBRA | 86.95 | -91.75; 265.65 | 0.283 |  |
|  | Intervention adherence (total score) | 76.93 | -49.88; 203.75 | 0.192 |  |
| PSMD | | | | | |
|  | PSMD at baseline | 0.83 | 0.56; 1.10 | **<0.001** |  |
|  | ∆LIBRA | -4.66*10^-6^ | -0.00001; 2.24*10^-6^ | 0.152 |  |
|  | Intervention adherence (total score) | 1.54*10^-6^ | -3.84*10^-7^; 3.45*10^-6^ | 0.099 |  |
| FW fraction | | | | | |
|  | FW at baseline | 1.17 | 0.76; 1.48 | **<0.001** |  |
|  | ∆LIBRA | 0.001 | -0.002; 0.004 | 0.360 |  |
|  | Intervention adherence (total score) | -0.0005 | -0.002; 0.001 | 0.522 |  |
| CBF | | | | | |
|  | CBF at baseline | 0.26 | -0.24; 0.76 | 0.254 |  |
|  | ∆LIBRA | 0.01 | -1.38; 1.41 | 0.981 |  |
|  | Intervention adherence (total score) | -1.01 | -3.90; 1.89 | 0.434 |  |
| *Cognitive performance* | | | | | |
| LDI | | | | | |
|  | LDI at baseline | 0.94 | -0.62; 2.49 | 0.196 |  |
|  | ∆LIBRA | -0.03 | -0.09; 0.03 | 0.301 |  |
|  | Intervention adherence (total score) | -0.008 | -0.02; 0.009 | 0.301 |  |
| REC | | | | | |
|  | REC at baseline | 0.33 | -0.94; 1.61 | 0.554 |  |
|  | ∆LIBRA | -0.02 | -0.05; 0.01 | 0.251 |  |
|  | Intervention adherence (total score) | 0.005 | -0.01; 0.02 | 0.524 |  |

Multivariable linear regression, outcomes: MRI-imaging markers and cognitive performance at follow-up, using n = 20 imputed datasets; Individual models for each imaging marker, respectively. All analyses controlling for baseline values of respective outcome, age, sex, and education (assessed using the Comparative Analysis of Social Mobility in Industrial Nations (CASMIN)-scale). Intervention adherence: total score, range: 10-28, higher values indicating better adherence. CBF: cerebral blood flow; CI: confidence interval; ECT: entorhinal cortex thickness; FW: free water; HCV: hippocampus volume; LDI: lure discrimination index; PSMD: peak width of skeletonized mean diffusivity; REC: recognition memory; WMH: white matter hyperintensity.

**Supplemental Table 5.** Change in individual LIBRA-factors from baseline to follow-up

| LIBRA factor | Total (n = 41; % (n)) | | IG (n = 16; % (n)) | | CG (n = 25; % (n)) | | *p* | |  |
| --- | --- | --- | --- | --- | --- | --- | --- | --- | --- |
|  | **BL** | **FU** | **BL** | **FU** | **BL** | **FU** | |  | |
| Hypertension | 61.0 (25) | 36.6 (15) | 87.5 (14) | 37.5 (6) | 44.0 (11) | 36.0 (9) | | 0.017 | |
| Hypercholesterolemia | 53.7 (22) | 53.7 (22) | 37.5 (6) | 37.5 (6) | 64.0 (16) | 64.0 (16) | | / | |
| Obesity | 26.8 (11) | 31.7 (13) | 37.5 (6) | 37.5 (6) | 20.0 (5) | 28.0 (7) | | 0.206 | |
| Physical inactivity | 63.4 (26) | 36.6 (15) | 68.8 (11) | 12.5 (2) | 60 (15) | 52.0 (13) | | 0.017 | |
| Diabetes | 68.3 (28) | 70.7 (29) | 56.3 (9) | 62.5 (10) | 76.0 (19) | 76.0 (19) | | 0.636 | |
| Renal dysfunction | 14.6 (6) | 9.8 (4) | 6.3 (1) | 6.3 (1) | 20.0 (5) | 12.0 (3) | | 0.224 | |
| Coronary heart disease | 7.3 (3) | 14.6 (6) | 6.3 (1) | 6.3 (1) | 8.0 (2) | 20.0 (5) | | / | |
| Depression | 9.8 (4) | 17.1 (7) | 0 | 12.5 (2) | 16.0 (4) | 20.0 (5) | | 0.246 | |
| Smoking | 7.5 (3) | 7.5 (3) | 12.5 (2) | 12.5 (2) | 4.2 (1) | 4.2 (1) | | / | |
| Low/moderate alcohol consumption | 8.3 (3) | 31.7 (13) | 0 | 31.3 (5) | 14.3 (3) | 32.0 (8) | | 0.834 | |
| Healthy diet | 56.1 (23) | 31.7 (13) | 37.5 (6) | 75.0 (12) | 68.0 (17) | 64.0 (16) | | 0.020 | |
| High cognitive activity | 41.0 (16) | 56.1 (23) | 46.7 (7) | 56.3 (9) | 37.5 (9) | 56.0 (14) | | 0.534 | |

BL: baseline; CG: control group; FU: follow-up; IG: intervention group; LIBRA: Lifestyle for Brain Health Index; *p* denotes significance of between-group difference in change from baseline to follow-up

**Supplemental Table 6.** Multivariable regression analyses assessing effects of changes in LIBRA-components on imaging markers at follow-up

| Outcome | EC thickness | | |  | HCV | | |
| --- | --- | --- | --- | --- | --- | --- | --- |
|  | **b (95% CI)** | **p** |  | | **b, 95% CI** | **p** |  |
| Predictors |  |  | **Predictors** | |  |  |  |
| ∆ Physical inactivity | 0.03 (-0.06; 0.12) | 0.471 | ∆ Physical inactivity | | 1.52 (-45.38; 48.43) | 0.947 |  |
| IG (ref: CG) | -0.04 (-0.13; 0.04) | 0.289 | IG (ref: CG) | | 18.01 (-63.44; 99.46) | 0.650 |  |
| ∆ Physical inactivity*IG | -0.04 (-0.19; 0.10) | 0.547 | ∆ Physical inactivity*IG | | -12.49 (-110.24; 85.25) | 0.793 |  |
| ∆ Obesity | 0.12 (0.03; 0.21) | **0.014** | ∆ Obesity | | -112.61 (-156.84; -68.38) | **<0.001** |  |
| IG (ref: CG) | -0.06 (-0.12; 0.01) | 0.076 | IG (ref: CG) | | 11.68 (-33.28; 56.63) | 0.594 |  |
| ∆ Obesity*IG | / |  | ∆ Obesity*IG | | / |  |  |
| ∆ Renal dysfunction | 0.02 (-0.05; 0.10) | 0.540 | ∆ Renal dysfunction | | 58.56 (8.00; 109.11) | **0.025** |  |
| IG (ref: CG) | -0.04 (-0.11; 0.04) | 0.294 | IG (ref: CG) | | 18.07 (-22.59; 58.73) | 0.365 |  |
| ∆ Renal dysfunction*IG | -0.13 (-0.25; -0.01) | **0.032** | ∆ Renal dysfunction*IG | | -16.98 (-90.92; 56.95) | 0.637 |  |
| ∆ Hypertension | -0.07 (-0.14; 0.01) | 0.077 | ∆ Hypertension | | -50.60 (-112.44; 11.24) | 0.103 |  |
| IG (ref: CG) | -0.10 (-0.24; 0.04) | 0.166 | IG (ref: CG) | | 15.44 (-38.19; 69.06) | 0.555 |  |
| ∆ Hypertension*IG | 0.13 (-0.04; 0.31) | 0.128 | ∆ Hypertension*IG | | 29.69 (-62.36; 121.74) | 0.509 |  |
| ∆ Healthy diet | -0.02 (-0.07; 0.04) | 0.551 | ∆ Healthy diet | | -14.46 (-74.90; 45.99) | 0.623 |  |
| IG (ref: CG) | -0.03 (-0.13; 0.08) | 0.616 | IG (ref: CG) | | 22.33 (-29.17; 73.82) | 0.377 |  |
| ∆ Healthy diet*IG | -0.05 (-0.21; 0.11) | 0.545 | ∆ Healthy diet*IG | | -10.22 (-94.53; 74.08) | 0.803 |  |
| ∆ Depression | -0.13 (-0.23; -0.03) | **0.011** | ∆ Depression | | 26.05 (-69.62; 121.72) | 0.577 |  |
| IG (ref: CG) | -0.08 (-0.16; -0.01) | **0.032** | IG (ref: CG) | | 16.71 (-26.41; 59.84) | 0.429 |  |
| ∆ Depression*IG | / |  | ∆ Depression*IG | | / |  |  |
| ∆ High cognitive activity | 0.03 (-0.08; 0.14) | 0.594 | ∆ High cognitive activity | | -40.58 (-101.48; 20.31) | 0.179 |  |
| IG (ref: CG) | -0.06 (-0.12; -0.01) | **0.034** | IG (ref: CG) | | 10.07 (-33.08; 53.22) | 0.632 |  |
| ∆ High cognitive activity*IG | 0.06 (-0.12; 0.23) | 0.487 | ∆ High cognitive activity*IG | | 19.06 (-70.68; 108.81) | 0.662 |  |
| ∆ Low/mod. alcohol consumption | 0.19 (0.08; 0.31) | **0.004** | ∆ Low/mod. alcohol consumption | | -6.93 (-122.02; 108.15) | 0.901 |  |
| IG (ref: CG) | -0.04 (-0.10; 0.02) | 0.208 | IG (ref: CG) | | 4.39 (-43.02; 51.80) | 0.849 |  |
| ∆ Low/mod. alcohol consumption*IG | -0.16 (-0.33; 0.01) | 0.062 | ∆ Low/mod. alcohol consumption*IG | | 68.64 (-99.76; 226.04) | 0.367 |  |
| ∆ Diabetes | 0.02 (-0.16; 0.19) | 0.845 | ∆ Diabetes | | 19.85 (-51.70; 91.39) | 0.566 |  |
| IG (ref: CG) | -0.05 (-0.14; 0.03) | 0.206 | IG (ref: CG) | | 16.92 (-33.22; 67.07) | 0.490 |  |
| ∆ Diabetes*IG | -0.01 (-0.33; 0.32) | 0.968 | ∆ Diabetes*IG | | -32.48 (-117.22; 52.27) | 0.432 |  |

**Supplemental Table 6 (continued).** Multivariable regression analyses assessing effects of changes in LIBRA-components on imaging markers at follow

| Outcome | WMH volume | | |  | PSMD | |
| --- | --- | --- | --- | --- | --- | --- |
|  | **b, 95% CI** | **p** |  | | **b, 95% CI** | **p** |
| Predictors |  |  | **Predictors** | |  |  |
| ∆ Physical inactivity | -159.50 (-937.68; 618.68) | 0.674 | ∆ Physical inactivity | | 0.00001 (-7.66*10^-6^; 0.00003) | 0.222 |
| IG (ref: CG) | 534.72 (-223.49; 1,292.93) | 0.157 | IG (ref: CG) | | 5.65*10^-6^ (-0.00002; 9.00*10^-6^) | 0.431 |
| ∆ Physical inactivity*IG | -241.41 (-1,597.15; 1,114.32) | 0.714 | ∆ Physical inactivity*IG | | -0.00002 (-0.00004; 8.60*10^-6^) | 0.200 |
| ∆ Obesity | -326.56 (-1,111.53; 458.40) | 0.396 | ∆ Obesity | | 7.88*10^-6^ (-7.65*10^-6^; 0.00002) | 0.303 |
| IG (ref: CG) | 290.26 (-396.82; 977.33) | 0.389 | IG (ref: CG) | | -9.72*10^-6^ (-0.00002; 4.49*10^-6^) | 0.169 |
| ∆ Obesity*IG | / |  | ∆ Obesity*IG | | / |  |
| ∆ Renal dysfunction | 289.23 (-522.74; 1,101.20) | 0.466 | ∆ Renal dysfunction | | -8.98*10^-6^ (-0.00003; 0.00002) | 0.478 |
| IG (ref: CG) | 321.50 (-511.92; 1,154.93) | 0.431 | IG (ref: CG) | | -0.00001 (-0.00003; 4.70*10^-6^) | 0.158 |
| ∆ Renal dysfunction*IG | -213.67 (-1,517.92; 1,090.57) | 0.736 | ∆ Renal dysfunction*IG | | 8.98*10^-6^ (-0.00002; 0.00004) | 0.563 |
| ∆ Hypertension | 441.37 (-51.66; 874.41) | 0.079 | ∆ Hypertension | | -2,16*10^-6^ (-0.00002; 0.00001) | 0.785 |
| IG (ref: CG) | 557.61 (-17.97; 1,133.19) | 0.057 | IG (ref: CG) | | -5.65*10^-6^ (-0.00002; 9.14*10^-6^) | 0.435 |
| ∆ Hypertension*IG | -803.58 (-1,613.09; 5.92) | 0.052 | ∆ Hypertension*IG | | -5.63*10^-6^ (-0.00003; 0.00001) | 0.561 |
| ∆ Healthy diet | -245.93 (-1,376.12; 884.25) | 0.655 | ∆ Healthy diet | | -3.19*10^-6^ (-0.00002; 0.00001) | 0.628 |
| IG (ref: CG) | -108.89 (-1,068.54; 850.76) | 0.815 | IG (ref: CG) | | -0.00001 (-0.00003; 1.00*10^-5^) | 0.277 |
| ∆ Healthy diet*IG | 933.48 (-553.72; 2,420.68) | 0.205 | ∆ Healthy diet*IG | | 6.37*10^-6^ (-0.00002; 0.00003) | 0.585 |
| ∆ Depression | 742.40 (-711.85; 2,196.64) | 0.300 | ∆ Depression | | -4.63*10^-6^ (-0.00003; 0.00002) | 0.729 |
| IG (ref: CG) | 404.04 (-383.63; 1,191.70) | 0.298 | IG (ref: CG) | | -0.00001 (-0.00003; 5.66*10^-6^) | 0.191 |
| ∆ Depression*IG | / |  | ∆ Depression*IG | | / |  |
| ∆ High cognitive activity | -450.04 (-1,530.35; 630.27) | 0.384 | ∆ High cognitive activity | | -1.70*10^-6^ (-0.00002; 0.00001) | 0.805 |
| IG (ref: CG) | 254.62 (-535.53; 1,044.76) | 0.509 | IG (ref: CG) | | -0.00001 (-0.00003; 4.04*10^-6^) | 0.142 |
| ∆ High cognitive activity*IG | 287.51 (-1,066.01; 1,641.04) | 0.656 | ∆ High cognitive activity*IG | | 0.00001 (-0.00001; 0.00004) | 0.285 |
| ∆ Low/mod. alcohol consumption | 82.32 (-799.09; 963.73) | 0.842 | ∆ Low/mod. alcohol consumption | | -0.00001 (-0.00004; 0.00002) | 0.386 |
| IG (ref: CG) | 301.08 (-478.00; 1,080.17) | 0.430 | IG (ref: CG) | | -0.00001 (-0.00003; 4.63*10^-6^) | 0.145 |
| ∆ Low/mod. alcohol consumption*IG | -18.14 (-1,430.83; 1,394.55) | 0.978 | ∆ Low/mod. alcohol consumption*IG | | 0.00003 (-0.00002; 0.0001) | 0.216 |
| ∆ Diabetes | 463.07 (-226.98; 1,153.12) | 0.174 | ∆ Diabetes | | 2.39*10^-6^ (-0.00003; 0.00003) | 0.864 |
| IG (ref: CG) | 411.52 (-356.10; 1,179.13) | 0.276 | IG (ref: CG) | | -0.00001 (-0.00003; 8.22*10^-6^) | 0.251 |
| ∆ Diabetes*IG | -687.32 (-2,332.15; 957.51) | 0.374 | ∆ Diabetes*IG | | 6.49*10^-6^ (-0.00003; 0.00005) | 0.731 |

**Supplemental Table 6 (continued).** Multivariable regression analyses assessing effects of changes in LIBRA-components on imaging markers at follow

| Outcome | FW fraction | |  | | CBF | | |  |
| --- | --- | --- | --- | --- | --- | --- | --- | --- |
|  | **b, 95% CI** | **p** | |  | | **b, 95% CI** | **p** | |
| Predictors |  |  | | **Predictors** | |  |  | |
| ∆ Physical inactivity | 0.01 (-0.01; 0.02) | 0.353 | | ∆ Physical inactivity | | 3.60 (-5.83; 13.03) | 0.435 | |
| IG (ref: CG) | 0.01 (-0.01; 0.25) | 0.495 | | IG (ref: CG) | | 4.20 (-4.78; 13.18) | 0.341 | |
| ∆ Physical inactivity*IG | -0.0003 (-0.03; 0.03) | 0.984 | | ∆ Physical inactivity*IG | | -1.28 (-13.59; 11.03) | 0.831 | |
| ∆ Obesity | -0.02 (-0.03; -0.01) | **0.005** | | ∆ Obesity | | -8.72 (-20.15; 2.71) | 0.127 | |
| IG (ref: CG) | 0.01 (-0.001; 0.02) | 0.084 | | IG (ref: CG) | | 5.14 (-0.76; 11.03) | 0.084 | |
| ∆ Obesity*IG | / |  | | ∆ Obesity*IG | | / |  | |
| ∆ Renal dysfunction | 0.01 (-0.0003; 0.03) | 0.054 | | ∆ Renal dysfunction | | -3.08 (-8.93; 2.77) | 0.284 | |
| IG (ref: CG) | 0.01 (0.001; 0.02) | **0.032** | | IG (ref: CG) | | 6.25 (-0.31; 12.82) | 0.061 | |
| ∆ Renal dysfunction*IG | -0.01 (-0.03; 0.01) | 0.230 | | ∆ Renal dysfunction*IG | | -12.06 (-26.14; 2.01) | 0.089 | |
| ∆ Hypertension | 0.0004 (-0.01; 0.01) | 0.945 | | ∆ Hypertension | | 8.89 (0.13; 17.64) | **0.047** | |
| IG (ref: CG) | 0.01 (-0.01; 0.02) | 0.281 | | IG (ref: CG) | | 4.11 (-3.07; 11.29) | 0.247 | |
| ∆ Hypertension*IG | 0.004 (-0.01; 0.02) | 0.574 | | ∆ Hypertension*IG | | -5.07 (-14.77; 4.62) | 0.288 | |
| ∆ Healthy diet | 0.003 (-0.007; 0.01) | 0.565 | | ∆ Healthy diet | | 6.25 (-0.71; 13.21) | 0.076 | |
| IG (ref: CG) | 0.004 (-0.01; 0.02) | 0.656 | | IG (ref: CG) | | 10.18 (0.17; 20.19) | **0.047** | |
| ∆ Healthy diet*IG | 0.009 (-0.01; 0.03) | 0.325 | | ∆ Healthy diet*IG | | -14.89 (-34.14; 4.37) | 0.123 | |
| ∆ Depression | 0.01 (-0.03; 0.05) | 0.519 | | ∆ Depression | | -17.23 (-30.48; -3.98) | **0.013** | |
| IG (ref: CG) | 0.01 (0.002; 0.02) | **0.020** | | IG (ref: CG) | | 1.03 (-5.34; 7.41) | 0.738 | |
| ∆ Depression*IG | / |  | | ∆ Depression*IG | | / |  | |
| ∆ High cognitive activity | -0.002 (-0.01; 0.01) | 0.734 | | ∆ High cognitive activity | | 9.75 (-0.39; 19.90) | 0.059 | |
| IG (ref: CG) | 0.01 (-0.002; 0.02) | 0.105 | | IG (ref: CG) | | 6.33 (-0.07; 12.73) | 0.052 | |
| ∆ High cognitive activity*IG | 0.001 (-0.02; 0.02) | 0.883 | | ∆ High cognitive activity*IG | | -12.79 (-29.37; 3.78) | 0.123 | |
| ∆ Low/mod. alcohol consumption | -0.01 (-0.02; 0.003) | 0.114 | | ∆ Low/mod. alcohol consumption | | 5.34 (-14.13; 24.80) | 0.573 | |
| IG (ref: CG) | 0.01 (-0.002; 0.02) | 0.102 | | IG (ref: CG) | | 5.83 (0.03; 11.63) | **0.049** | |
| ∆ Low/mod. alcohol consumption*IG | -0.005 (-0.03; 0.02) | 0.663 | | ∆ Low/mod. alcohol consumption*IG | | -7.28 (-28.18; 13.62) | 0.476 | |
| ∆ Diabetes | -0.003 (-0.02; 0.01) | 0.692 | | ∆ Diabetes | | 6.55 (-11.44; 24.54) | 0.451 | |
| IG (ref: CG) | 0.01 (-0.005; 0.02) | 0.178 | | IG (ref: CG) | | 8.36 (-0.14; 16.87) | 0.054 | |
| ∆ Diabetes*IG | -0.003 (-0.05; 0.04) | 0.881 | | ∆ Diabetes*IG | | -20.69 (-41.97; 0.59) | 0.056 | |

Multivariable linear regression, outcomes: MRI-imaging markers at follow-up, using n = 20 imputed datasets. Individual models for changes (∆) in each component of LIBRA, respectively. All analyses controlling for baseline values of respective outcome, age, sex, and education (assessed using the Comparative Analysis of Social Mobility in Industrial Nations (CASMIN)-scale). CBF: cerebral blood flow; CG: control group; CI: confidence interval; ECT: entorhinal cortex thickness; FW: free water; HCV: hippocampus volume; IG: intervention group; PSMD: peak width of skeletonized mean diffusivity; WMH: white matter hyperintensity.

We tested whether baseline LIBRA or change in LIBRA were associated with average cortical thickness (CT) in 68 regions from the Desikan-Killiany atlas. We calculated the mean over the imputations to extract average LIBRA at baseline and average change in LIBRA and conducted the same models, adjusting for age, sex, education and baseline CT for change analyses.

There were no statistically significant associations for LIBRA at baseline, the interaction of group and ΔLIBRA or ΔLIBRA alone when correcting for multiple comparisons using the Benjamini-Hochberg approach.

Descriptively, the strongest negative associations between baseline LIBRA and cortical thickness were found for the cuneus bilaterally (left: β = -0.02, SE = 0.007, *p* = 0.0067, *p_adj* = 0.38, right: β = -0.015, SE = 0.007, *p* = 0.023, *p_adj* = 0.38) and the pericalcarine cortex on the right hemisphere (β = -0.022, SE = 0.009, *p* = 0.021, *p_adj* = 0.38) (see top row for Supplemental Figure 1). A positive association was found for the left caudal anteriorcingulate cortex (β = 0.033, SE = 0.013, *p* = 0.014, *p_adj* = 0.38, right).

For the association of ΔLIBRA and the interaction of ΔLIBRA and group, the strongest associations were found for the pericalcarine cortex on the left hemisphere where increase in LIBRA was associated with reduced CT in this area (β = -0.017, SE = 0.006, *p* = 0.0069, *p_adj* = 0.47) but not in the intervention group (β = 0.025, SE = 0.011, *p* = 0.02, *p_adj* = 0.99).

**Supplemental Figure 1.** Regression coefficients for CT in Desikan-Killiany regions for upper row: baseline LIBRA, middle row: ΔLIBRA and lower row: the interaction of group and ΔLIBRA. White/black indicate a positive/negative association of LIBRA and ΔLIBRA with CT. No association was significant after multiple comparison correction.


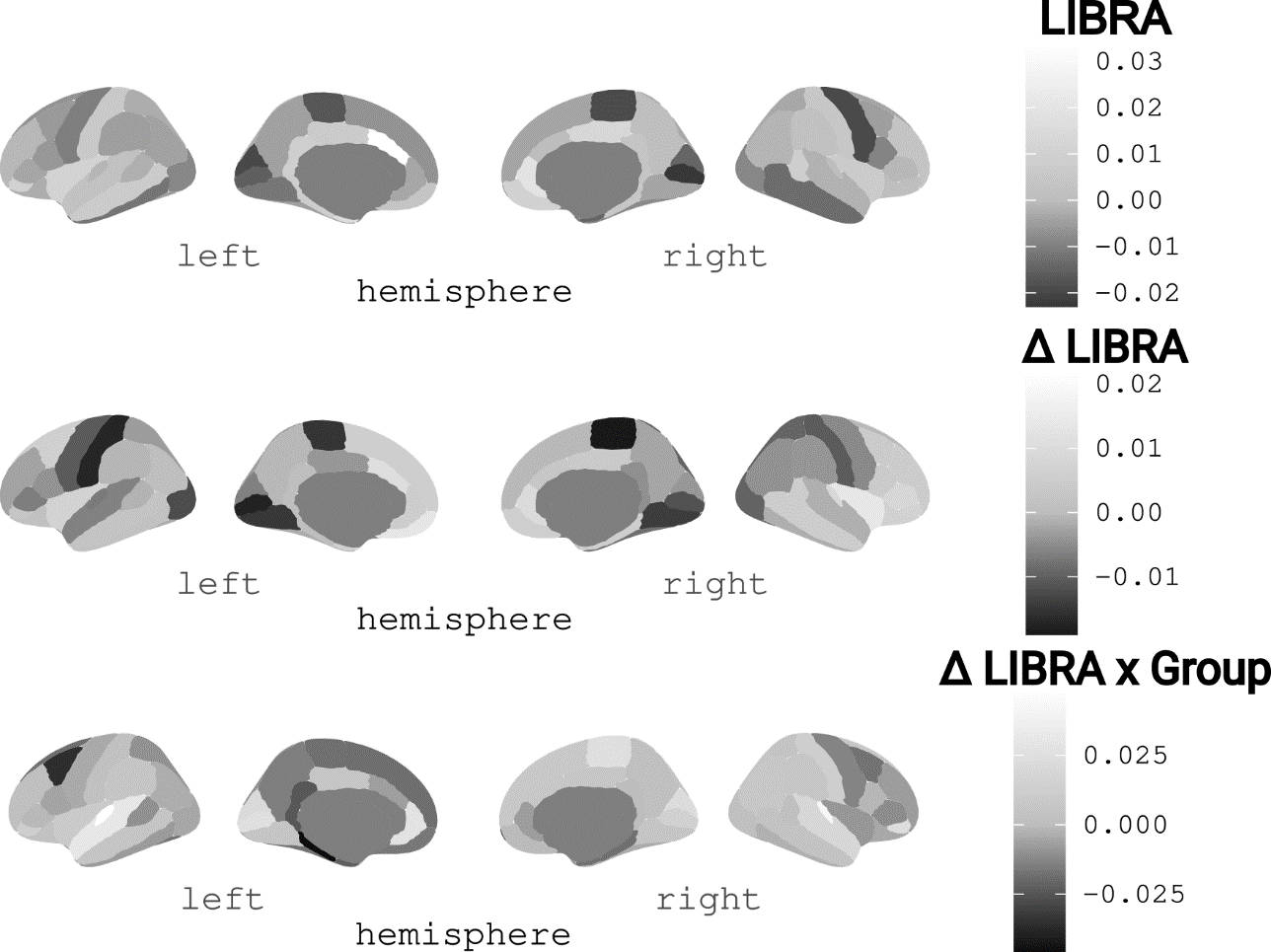

Supplement: sj-docx-1-alz-10.1177_13872877251414423 - Supplemental material for Evaluating MRI correlates of lifestyle-based dementia risk reduction: Results from the AgeWell.de imaging study [file sj-docx-1-alz-10.1177_13872877251414423.docx]
